# Burden of fungal asthma in Africa: A systematic review and meta-analysis

**DOI:** 10.1371/journal.pone.0216568

**Published:** 2019-05-16

**Authors:** Richard Kwizera, Joseph Musaazi, David B. Meya, William Worodria, Freddie Bwanga, Henry Kajumbula, Stephen J. Fowler, Bruce J. Kirenga, Robin Gore, David W. Denning

**Affiliations:** 1 Infectious Diseases Institute, College of Health Sciences, Makerere University, Kampala, Uganda; 2 Makerere University Lung Institute, College of Health Sciences, Makerere University, Kampala, Uganda; 3 Department of Medicine, School of Medicine, College of Health Sciences, Makerere University, Kampala, Uganda; 4 Mulago National Referral Hospital, Kampala, Uganda; 5 Department of Medical Microbiology, School of Biomedical Sciences, College of Health Sciences, Makerere University, Kampala, Uganda; 6 Division of Infection, Immunity and Respiratory Medicine, School of Biological Sciences, Faculty of Biology, Medicine and Health, The University of Manchester; NIHR Biomedical Research Centre, Manchester University Hospitals NHS Foundation Trust, Manchester, United Kingdom; 7 Cambridge University Hospitals NHS Foundation Trust, Cambridge, United Kingdom; 8 The National Aspergillosis Centre, Wythenshawe Hospital, The University of Manchester, Manchester Academic Health Science Centre, Manchester, United Kingdom; Clinic for Infectious and tropical diseases, Clinical centre of Serbia, SERBIA

## Abstract

**Background:**

Asthma is one of the neglected diseases in Africa with a high prevalence. Allergic fungal diseases have been reported to complicate asthma progression and treatment outcomes. However, data about fungal asthma and its associated complications are limited in Africa. We aimed to estimate the burden of fungal asthma among adults and children in Africa using a systematic review.

**Methods:**

We first engaged the Institute for Health Metrics and Evaluation (IHME) to highlight the trend in morbidity and mortality attributed to asthma in Africa. We then searched PubMed, HINARI and Google Scholar for all studies of any design focusing on fungal asthma in any African country. Languages were restricted to English and French, but not year of publication. We estimated the weighted prevalence of allergic fungal infections among asthmatics with a 95% CI and pooled the results using a random effects model. This study is registered with PROSPERO, number CRD42019117319.

**Results:**

The IHME data showed that there has been a gradual increase in morbidity and mortality due to asthma in African adults with a prevalence of 4%. Our search retrieved 5233 citations. We retained 20 studies that met our selection criteria. These were from 13 African countries published between 1967 and 2018. There were eight cross-sectional studies and twelve review articles. The average asthma prevalence in Africa was 6% from these studies. The prevalence of fungal sensitisation was relatively high (3–52%) in the asthmatic population with an average of 28% and a pooled estimate of 23.3%, mostly due to *Aspergillus* species. Prevalence of Allergic bronchopulmonary apsergillosis was estimated at 1.6–21.2%. Diagnosis of fungal allergy was mostly made by skin prick tests. There was no data on the use of medication to manage fungal asthma. None of the studies evaluated the association between fungal allergy and asthma severity. Data were lacking in children.

**Conclusion:**

There is a high prevalence of fungal sensitization among Africans with asthma. Fungal asthma is a significant problem in Africa but there remains a paucity of data on the epidemiology and associated complications. There is urgent need for national epidemiological studies to estimate the actual burden of fungal asthma in Africa.

## Introduction

The global prevalence of asthma ranges from 1% to 18% in different studies [1–4], with national estimates ranging from 1.7% to 53% in different countries and at different ages [5,6]. In Africa, asthma is one of many neglected diseases and its prevalence is estimated with an average of 12% [6,7] and national estimates ranging from 2% to 53% among individuals of age ranging from <2 to 64 years [5,6,8–13]. Most surveys in Africa have been conducted among children aged 5–14 years and few studies in adults [6,14]. Urban dwellers are more affected with asthma than people living in rural areas [14]. However, a systematic review by Anandan *et al* [12] revealed that although asthma prevalence had been reported in some parts of Africa, no serial data has been reported yet. It was also highlighted that there are limited data on asthma trends in Africa and none using serial cohort designs [12]. Furthermore, it is estimated that up to 10% of asthma patients globally have severe asthma [15,16], and various factors have been proposed as underlying severe asthma among Africans [6,17–20].

Fungal exposure (related to dampness in housing) may precipitate the development of asthma [21–25]. Fungal sensitisation (or allergy) is known to worsen asthma control, leading to asthma attacks [26], additional need for corticosteroids, hospitalisation [27] and, in the case of *Aspergillus fumigatus*, increased rates of bronchiectasis [28–30]. Many airborne fungi are linked to poor asthma control but *Aspergillus* species are the strongest candidates [31], especially *Aspergillus fumigatus* [32]. Fungal sensitisation (or allergy) can be diagnosed by skin prick test (SPT) or fungal specific IgE immunoassays [28,32,33].

The clinical phenotypes of asthma linked to *Aspergillus* sensitisation includes allergic bronchopulmonary aspergillosis (ABPA) and/or severe asthma with fungal sensitisation (SAFS) [28] since some ABPA patients will have severe asthma, with or without bronchiectasis. These two pulmonary disorders worsen asthma status and are strongly associated with reduced lung function and poor treatment outcomes despite apparently optimal care for asthma [30,32,34–36]. Excess corticosteroids are a common consequence of poor control in these patients, with their well known plethora of long term side effects. *Aspergillus*, continuously present in the airway [37], in addition to sensitisation, probably drives the immunopathology [38]. Similarly, in ABPA a predominant cellular T-helper cell (Th)-2 type response with high IgE and eosinophil counts, occurs in response to *Aspergillus* persisting in the airways [39,40].

Both ABPA and SAFS respond to oral itraconazole and voriconazole therapy, both now listed as Essential Medicines by the WHO [32,41]. Given that anti-IgE therapy with omaluzimab is not approved for ABPA, and all monoclonal therapies for asthma are currently vastly too expensive for the majority of patients in low and middle income countries, antifungal therapy is an attractive option, if patients can be readily identified.

ABPA, SAFS and other allergic diseases associated with fungal sensitisation in asthma are now simply referred to as ‘fungal asthma’, partly for simplicity, and partly because of many overlapping clinical features. However, despite published work about fungal asthma, data on its burden and associated factors remains scanty in Africa. The aim of this study was to systematically review literature on the burden of fungal asthma in Africa to highlight the gap in published data; noting information such as prevalence, diagnosis, treatment and the effect of fungal sensitisation on the severity of asthma. However, in order to understand the context of the burden of fungal allergy in asthma, we first estimated trends in asthma morbidity and mortality in Africa using data available from the Institute for Health Metrics and Evaluation (IHME) [42].

## Materials and methods

### Morbidity and mortality due to asthma in Africa from IHME

We first searched the IHME to review the trend in the morbidity and mortality attributed to asthma in Africa. For this query, we considered all ages (adults and children) and both sexes (female and male). We engaged the Global Health Data Exchange tool in the same database (http://ghdx.healthdata.org/gbd-results-tool) using “asthma” as the cause. We searched results ranging from 1990 to 2017 to observe any trends.

### Study designs, inclusion and exclusion criteria for the systematic review articles

This was a systematic review and meta-analysis performed according to PRISMA checklist (S1 Checklist). The systematic review protocol (S1 Protocol) was registered in the PROSPERO international prospective register of systematic reviews (No: CRD42019117319) (https://www.crd.york.ac.uk/PROSPEROFILES/117319_PROTOCOL_20181120.pdf). In the systematic review, we aimed to include all studies of any design focusing on fungal asthma in any African country, highlighting prevalence, diagnosis, treatment and the effect of fungal sensitisation on the severity of asthma. We restricted the languages to English and French since they are the main national languages in Africa. There was no restriction on year of publication. We planned to exclude all case reports/ series, studies about fungal sensitisation in populations other than asthma, studies done outside Africa and studies done in animal models. For this review, we defined fungal sensitisation as a positive fungal specific SPT or an elevated fungal specific IgE antibody titre.

### Search strategy for the systematic review

To capture as many relevant citations as possible, a PubMed electronic search was executed to identify primary studies addressing fungal asthma in Africa. In the first search, we used the term “Africa” AND other individual key words, such as, fungal sensitisation; *Aspergillus* sensitisation; fungal allergy; fungal infections; fungal asthma; severe asthma with fungal sensitisation; allergic bronchopulmonary aspergillosis; severe asthma and burden of fungal infections. In the second search, we replaced the word “Africa” with specific names for each of the individual 54 African nations but kept all the other key words. Furthermore, we repeated these two searches in HINARI and Google scholar to provide more references.

### Review of studies for the systematic review

A database was created from the electronic searches and kept in EndNote X7 programme while restricting entry of duplicate citations. Two reviewers (RK and JM) screened the citations using title and abstract without blinding to capture relevant studies. The two review authors independently assessed the risk of bias in included studies by examining the raw data (if available), completeness of outcome data and any other problems that could produce a high risk of bias, such as selective outcome reporting and insufficient blinding. Disagreements between the two review authors over the risk of bias in particular studies was resolved by discussion, with involvement of a third review author where necessary. The database was then screened again using full text for each study to include only relevant articles. We only included studies addressing the burden of fungal sensitisation, SAFS or ABPA in patients with asthma in any African country.

### Data summary for the systematic review

Data from the final studies were summarised in an excel spreadsheet, recording information such as; title, first author, year of publication, country, study type, sample size, population, prevalence of fungal sensitisation, prevalence of ABPA, prevalence of SAFS, diagnosis of fungal allergy, factors associated with fungal allergy, fungal allergy *vs* severity of asthma and treatment of fungal allergy. This was later transferred to STATA version 14 (STATA, College Station, Texas) for meta-analysis.

### Statistical analysis

Data were analysed using STATA version 14 (STATA, College Station, Texas). Statistical/ meta-analysis aimed to determine pooled prevalence of fungal sensitisation, ABPA and SAFS in asthma among Africans. For studies which had prevalence for more than one fungus, an average was considered. Meta-analysis was performed using metaprop function that pools prevalence estimates and computes exact binomial and score test-based confidence intervals [43]. The pooled prevalence and confidence intervals of fungal sensitisation, ABPA and SAFS in the individual studies were calculated using a random effects model. Heterogeneity chi-square test was used to assess the level of variation of prevalence across studies. Results were presented on forest plots.

## Results

### Estimates from the IHME

#### Burden of asthma in Africa from the IHME

In 2017, the population of Africa was estimated at 1.2 billion people (https://www.populationpyramid.net/africa/2017/). Considering both adults and children of both sexes, our query in the IHME database showed that in the year 2017, the overall prevalence of asthma in Africa was estimated at 4.2% (range: 3.5–4.8) translating into 50,668,000 cases (range: 42,981,000–58,739,000). This was similar to the average prevalence (6%, n = 13) obtained from the studies included in our review.

However, according to the IHME, there was variation in the prevalence of asthma in Africa by age, i.e. 5.0% (8,770,000 cases) for children under 5 years, 5.7% (17,632,000 cases) for 5–14 years, 2.9% (17,484,000 cases) for 15–49 years, 4.7% (5,047,000 cases) for 50–69 years and 7.0% (1,735,000 cases) for 70+ years [42]. Considering sub-Saharan Africa alone, the prevalence of asthma was 4.6% (7,270,000 cases) for children under 5 years, 5.5% (15,167,000 cases) for 5–14 years, 2.7% (13,388,000 cases) for 15–49 years, 3.9% (3,232,000 cases) for 50–69 years and 5.8% (1,097,000 cases) for 70+ years.

In addition, without using the IHME, and considering that 10% of asthmatics globally have severe asthma based on a previously described model [16], we estimated the prevalence of severe asthma among the included studies to range from 0.2% to 1.5% with an average of 0.6% based on this model (Table 1).

**Table 1:** Studies describing the burden of fungal asthma in Africa.

Study, year, reference, Country	Study type	Sample size	Population	Prevalence of Asthma in population (x)	Estimated prevalence of severe Asthma (10%x)^#^	Prevalence of fungal sensitisation in asthma	Weighted estimate for fungal sensitisation % (95%CI)^¶^	Prevalence of ABPA in asthma	Prevalence of SAFS in asthma	Diagnosis of fungal allergy/ atopy
**el-Hefny et al, 1967** [44] **Egypt**	Cross sectional	645	Asthmatics	-	-	Asp-41.8%, Alter-40.3%, Penic-33.4%	38.4% (34.8, 42.3)	-	-	SPT
**Benatar, 1977** [45] **South Africa**	Cross sectional	258	Adult asthmatics	-	-	27.0%	26.7% (21.7, 32.5)	1.9%	-	SPT, Precipitins, Eosinophil
**Benatar et al, 1980** [46] **South Africa**	Cross sectional	500	Asthmatics	-	-	22.0%	22.0% (15.6, 25.8)	2.6%	-	SPT, Precipitins
**Alshishtawy et al, 1991** [47] **Egypt**	Cross sectional	68	Adult asthmatics	-	-	4.4%	2.9% (0.8, 10.1)	-	-	TIgE-ELISA, Fungal IgE- RAST
**Baatjies et al, 2009** [48] **South Africa**	Cross sectional	517	Bakery workers	Occupational asthma- 13.0%, Work aggravated asthma- 3.0%	1.3% 0.3%	MM- 7.0%, (Clad, Alter, Fusa) AF- 3.0%	4.8% (3.3, 7.0)	-	-	SPT, Fungal IgE
**Hasnain et al, 2012** [49] **Sudan**	Cross sectional	50	Allergic respiratory diseases	-	-	AF- 40.0% Clad- 42.0% Alter- 38.0%	40.0% (27.6, 53.8)	-	-	SPT
**Oluwole et al, 2013** [50] **Nigeria**	Cross sectional	1736	High school children	8.0%	0.8%	MM- 11.0%	10.9% (9.6, 12.5)	-	-	SPT
**Sabry et al, 2016** [51] **Egypt**	Cross sectional	52	Moderate & severe asthma	-	-	AF- 52.0%	51.9% (38.7, 64.9)	21.2%	-	SPT
**Denning et al, 2013** [16] Africa*	Review	NA	Adult asthmatics	-	-	-	NA	419,000 patients*	-	NA
**Oladele et al, 2014** [52] **Nigeria**	Review	NA	Adult asthmatics	15.2%	1.5%	-	NA	2.5%	3.3%	NA
**Badiane et al, 2015** [53] **Senegal**	Review	NA	Adult asthmatics	5.0%	0.5%	0.2%	NA	2.5%	3.3%	NA
**Faini et al, 2015** [54] **Tanzania**	Review	NA	Adult asthmatics	3.1%	0.3%	0.1%	NA	2.5%	3.3%	NA
**Parkes et al, 2015** [55] **Uganda**	Review	NA	Adult asthmatics	4.4%	0.4%	-	NA	2.5%	3.3%	NA
**Guto et al, 2016** [56] **Kenya**	Review	NA	Adults	3.1%	0.3%	-	NA	2.5%	3.3%	NA
**Chekiri-Talbi et al, 2017** [57] **Algeria**	Review	NA	Adult asthmatics	3.1%	0.3%	0.1%	NA	2.5%	3.3%	NA
**Zaki et al, 2017** [58] **Egypt**	Review	NA	Adult asthmatics	9.4%	0.9%	-	NA	2.5%	3.3%	NA
**Bamba et al, 2018** [59] **Burkina Faso**	Review	NA	Adult asthmatics	2.3%	0.2%	-	NA	2.5%	3.3%	NA
**Kalua et al, 2018** [60] **Malawi**	Review	NA	Adult asthmatics	4.7%	0.5%	-	NA	2.5%	3.3%	NA
**Mandengue et al, 2018** [61] **Cameron**	Review	NA	Adult asthmatics	2.7%	0.3%	-	NA	2.5%	3.3%	NA
**Sacarlal et al, 2018** [62] **Mozambique**	Review	NA	Adult asthmatics	4.7%	0.5%	-	NA	2.5%	3.3%	NA

Data presented are summaries of the studies that were identified describing the burden of allergic fungal asthma in Africa. ABPA = allergic bronchopulmonary aspergillosis, SAFS = severe asthma with fungal sensitisation, Asp = *Aspergillus* species, AF = *Aspergillus fumigatus*, Alter = *Alternaria* species, Penic = *Penicillium* species, Clad = *Cladosporium* species, Fusa = *Fusarium*, MM = mould mix, SPT = skin prick test, TIgE = total Immunoglobulin E, ELISA = enzyme-linked immunosorbent assay, RAST = radioallergosorbent test. NA = not applicable, - = missing data,

* One study was a review modelling the global burden of ABPA with asthma, estimating its burden in Africa.

^#^ Estimated that 10% of asthma patients have severe asthma.

^¶^ Weighted estimates are from a random effects model based on the variance in each individual study.

#### Morbidity and mortality attributed to asthma in Africa from the IHME

**Fig 1:**
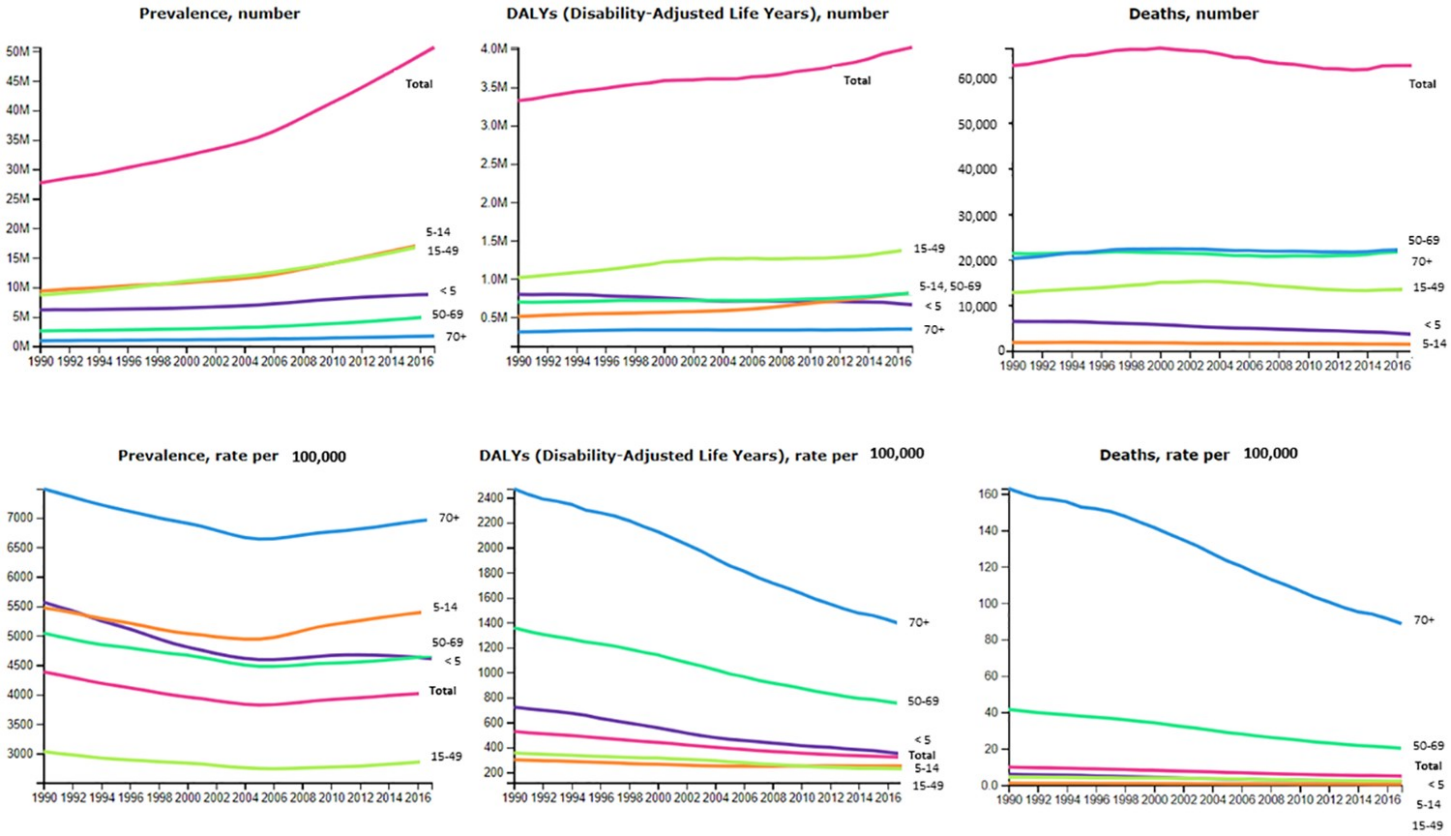
Trends in morbidity and mortality due to asthma in Africa. There has been a gradual increase in the prevalence, deaths and disability-adjusted life years attributed to asthma among adults in Africa over the years (source: IHME; Seattle, WA) [42]. Each line is a separate age category (years).

Our query in the IHME database for adults and children showed that in the year 2017 the overall observed mortality due to asthma in Africa was 0.7% (0.6–0.9) translating into 62,544 deaths (range: 50,665–74,430). However, mortality varied by age, i.e. 0.1% (3,617 deaths) among children under 5 years, 0.5% (1,437 deaths) for 5–14 years, 0.7% (13,546 deaths) for 15–49 years, 1.2% (21,876 deaths) for 50–69 years and 1.2% (22,066 deaths) for 70+ years. Similarly, the overall number of DALY’s were 4,020,082 years (range: 3,238,784–4,972,721) in the same year [42]. We then plotted these parameters from the year 1990 to 2017 to observe the trend (Fig 1). The IHME data indicate that the prevalence and DALY’s attributed to asthma in Africa have gradually increased among adults from 1990 to 2017. However, these results showed a gradual decrease in mortality among children less than fifteen years.

### Estimates from the systematic review

#### Search results

**Fig 2:**
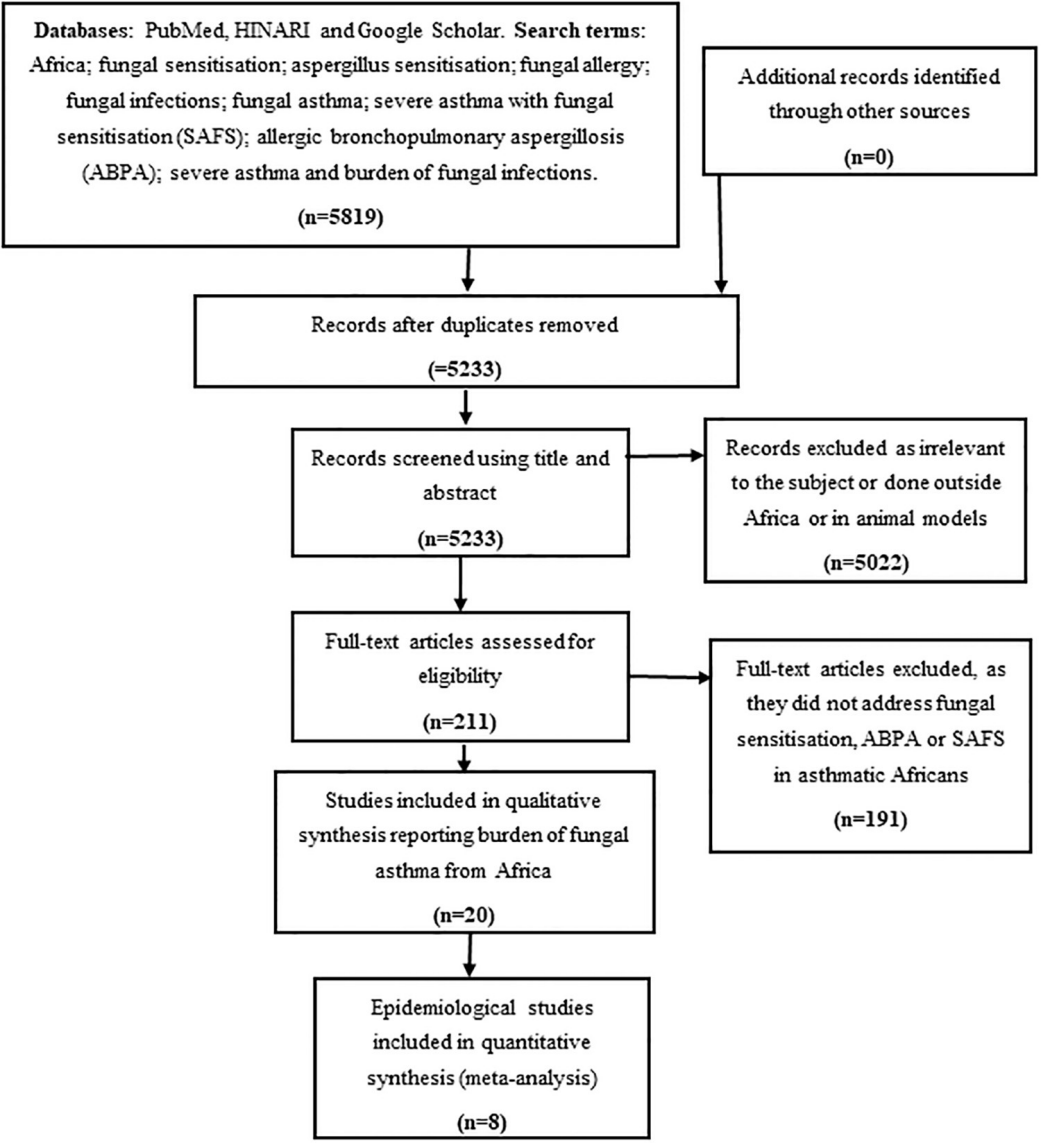
Citation selection process for the systematic review. Twenty studies were found describing the burden of fungal asthma in Africa. Eight were epidemiological studies and the twelve were review articles.

Our initial electronic database search for the systematic review in PubMed, HINARI and Google Scholar (hereafter referred to as electronic search) retrieved 5819 citations. We then removed duplicates and remained with 5233 citations from which relevant studies were selected for the review. Their potential relevance was examined using a title and abstract screening to remove studies that were clearly not related to the topic. 5022 citations were excluded as irrelevant to the subject. The full papers of the remaining 211 citations were assessed to select those that included data about fungal sensitisation, ABPA or SAFS in asthmatic Africans; highlighting any information on prevalence, diagnosis, treatment and the effect of fungal sensitisation on the severity of asthma. These criteria excluded 191 studies and left 20 studies [16,44–62] that were included in the final analysis. Of these 20 studies, only eight had enough data for a meta-analysis (Fig 2).

#### Summary of studies

**Fig 3:**
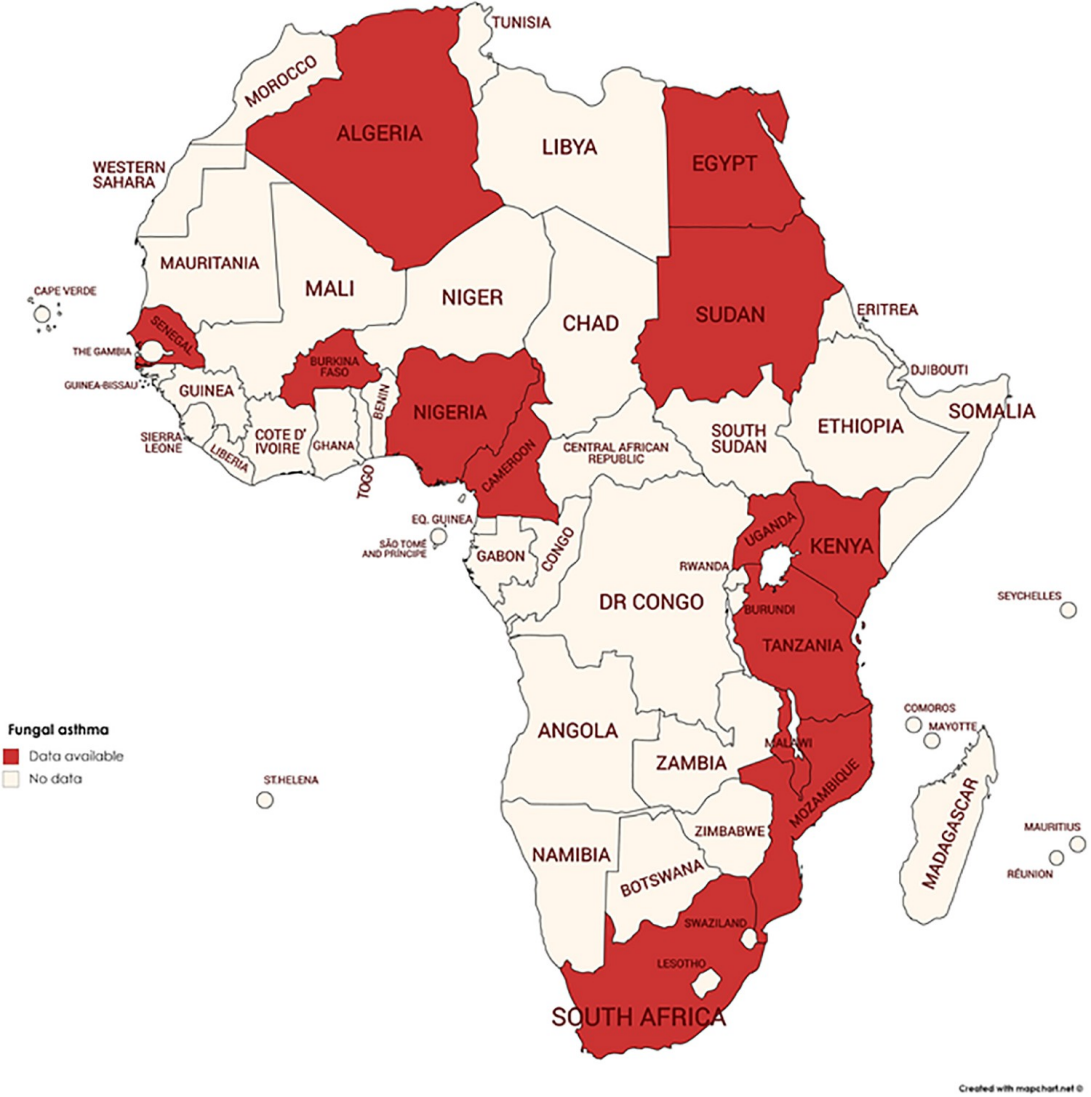
Distribution of studies describing burden of fungal asthma in Africa. Twenty studies were found from thirteen (out of 54) African countries. The shaded areas represent countries with data from epidemiological study and/or review article. Map was created and reprinted from [https://mapchart.net/africa.html#] under a CC BY license.

The 20 studies came from thirteen (13/54) African countries (Fig 3) including Egypt [44,47,51,58], South Africa [45,46,48], Senegal [53], Uganda [55], Algeria [57], Tanzania [54], Kenya [56], Sudan [49], Nigeria [50,52], Burkina Faso [59], Malawi [60], Cameroon [61] and Mozambique [62]. One study was a review modelling the global burden of ABPA with asthma, estimating its burden in Africa as well [16]. These were published between 1967 and 2018; with 16/20 (80%) of the studies published recently between 2009 and 2018, while the rest (4/20) were published between 1967 and 1991 (Table 1). No studies were found between 1992 and 2008. Eight of the studies were cross-sectional while twelve were review articles that used modelling to estimate the burden of fungal asthma.

#### Burden of fungal sensitisation in asthma

From the electronic search, only eleven studies described the prevalence of fungal sensitisation in adult asthmatics and none among children on the African continent. The prevalence of fungal sensitisation among asthmatics varied widely ranging from 3% to 52% [44–51,53,54,57] with an average of 28%. *Aspergillus* species were the most prevalent cause (3–52%) [44,48,49,51]. This was followed by *Alternaria* species (5.6–40.3%), *Cladosporium* species (4.2–42%) and the mould mix (7–11%).

**Fig 4:**
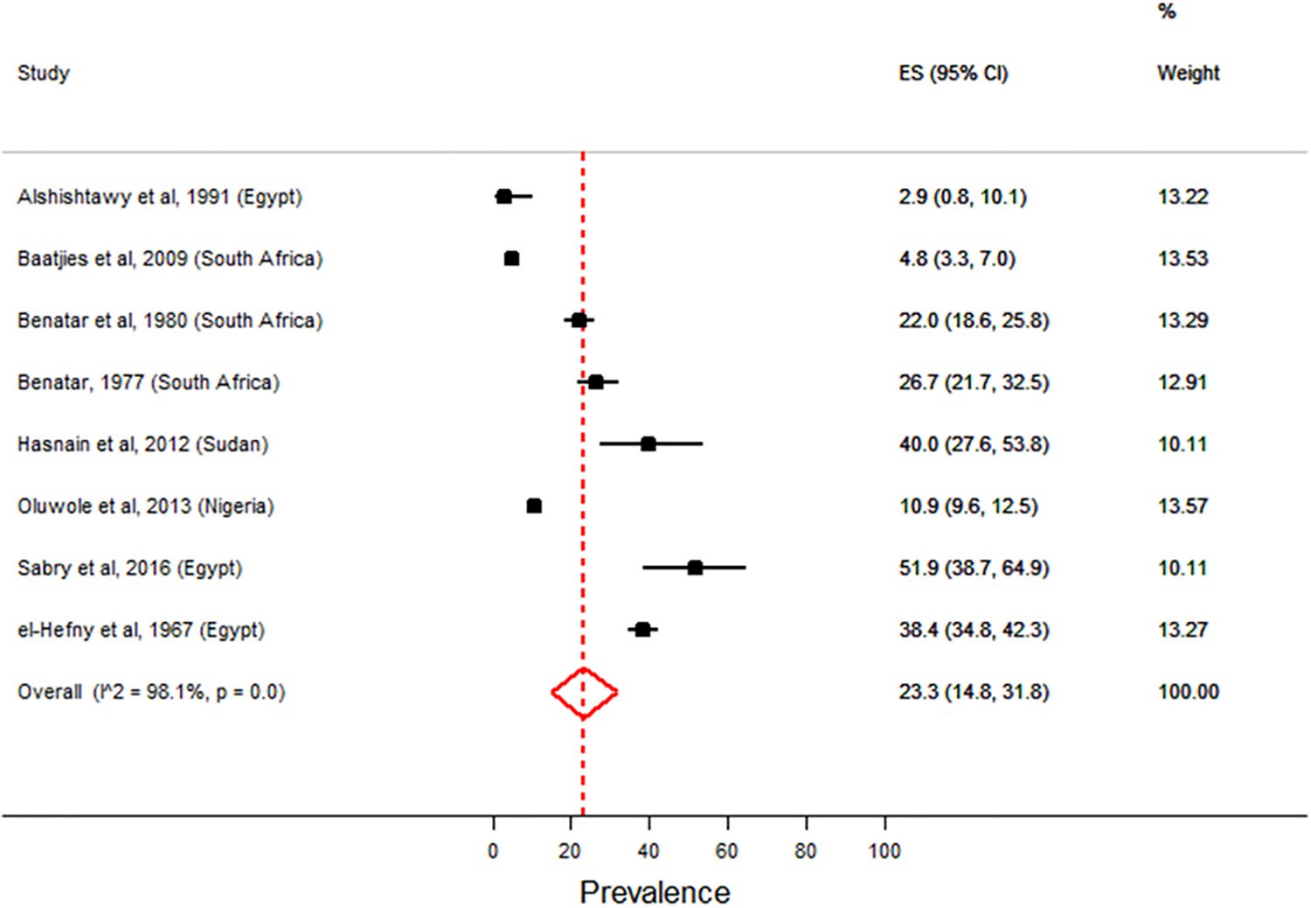
Forest plot showing meta-analysis on prevalence of fungal sensitisation. There was a lot of variation in the prevalence of fungal sensitisation across the eight studies included in the meta-analysis (p-value <0.01). Broken vertical line indicates the combined (overall) estimates. ES (95% CI) denotes weighted estimates of prevalence and their 95% confidence intervals based on the variance in each individual study.

Only the 8/20 cross-sectional studies had enough data to perform a meta-analysis for fungal sensitisation. Using a random effects model, the weighted estimates for fungal sensitisation ranged from 2.9 to 51.9% with a pooled prevalence estimate of 23.3% (CI: 14.8 to 31.8, n = 8 studies). There was a lot of variation in the prevalence of fungal sensitisation across the eight cross-sectional studies included in the meta-analysis (heterogeneity test; p-value <0.01) (Fig 4). The variation in the prevalence estimates attributable to heterogeneity was 98.1%. Only two studies [49,51] had very high variability in estimates; probably attributed to small sample sizes. Four of the studies had low prevalence estimates (<25%) while the remaining four studies had high prevalence estimates (>25%).

Diagnosis of fungal allergy was made using skin prick test in 7/8 cross-sectional studies. Of these, one study added fungal specific IgE (fungal α-amylase IgE) [48] while two studies added fungal precipitins test [45,46]. One study from Egypt used total serum IgE together with fungal specific IgE using the radioallergosorbent test (RAST) [47]. The rest of the studies were reviews of literature.

Only two studies [46,56] reported factors associated with fungal allergy. These pointed out associations with onset of asthma under age of 10 years, positive family history of atopy and proximity of garbage dumping sites. None of the studies evaluated the association between fungal allergy and asthma severity. None of the studies described fungal asthma in children.

#### Burden of ABPA in asthma

From the electronic search, only fifteen studies reported the prevalence of ABPA in asthma, in Africa. However, 12/15 studies reporting the estimates for ABPA were review articles without original data. All these reviews recommended an urgent need for national epidemiological studies to validate these estimates, since there are no reliable data on ABPA in Africa.

The prevalence of ABPA ranged from 1.6% to 21.2% among the studies included [45,46,51–62], most of which were reviews using modelling and published between 2013 and 2018. Due to limited published data on fungal disease epidemiology in Africa, the prevalence of ABPA was mostly estimated at 2.5% in all these reviews [52–62] based on a previously described model by *Denning et al*. [16] on the burden of ABPA among adults with asthma, including one study from South Africa. This model also estimated that there were 419,000 adult cases (range: 117,000 to 587,000) of ABPA in Africa. In 1977, Benatar *et al* estimated the prevalence of ABPA among adult asthmatics in South Africa at 1.9% (5/258) [45]. Similarly, in 1980 this group estimated the prevalence of ABPA in the same population to be 2.6% [46]. In 2016, Sabry *et al* studied adult patients with moderate and severe asthma in Egypt cross-sectionally and estimated the prevalence of ABPA at 21.2% in this selected cohort [51].

There were insufficient data to perform a meta-analysis for estimates of ABPA in Africa. For the three cross-sectional studies that described patients with ABPA [45,46,51], the diagnostic criteria was based on history of asthma symptoms, positive *Aspergillus* SPT, elevated eosinophil count, shadow of chest x-ray and positive precipitins test. Only one recent study included total IgE and chest CT scan [51]. Data were lacking on management for ABPA in Africa. Similarly, none of the studies evaluated the association between ABPA and asthma severity. The factors associated with ABPA remain unknown in the African context, including lack of any genetic or exposure studies, for example. There were no data about mortality related to ABPA in Africa. Data about the burden of ABPA in children was lacking in Africa.

#### Burden of SAFS in asthma

Eleven studies estimated the prevalence of SAFS from the electronic search. However, similar to ABPA, all estimates for SAFS in Africa were from country estimates of the burden of fungal disease [52–62] based on the model proposed by *Denning et al*. [16]. SAFS was estimated at 3.3% in all reviews. This model suggests that, “10% of asthma patients globally have severe asthma, and of these, 33% have been shown to be sensitised to one or more fungi”.

Just as for ABPA, there were insufficient data to perform a meta-analysis for estimates of SAFS in Africa, and whether asthma-related mortality is related to SAFS remains unknown in Africa. There are no published data regarding SAFS in children in Africa.

### Combined estimate for the burden of fungal asthma in Africa

In this section, we tried to estimate the burden of fungal asthma by combining results from both IHME and systematic review. From the IHME data, we used the “15–49 years” group to represent the population of adult asthmatics in Africa. If there were 17,484,000 cases of adult asthma with an estimated prevalence of ABPA of 2.5% and SAFS of 3.3% among adult asthmatics, we estimated that approximately 437,000 adult asthmatics had ABPA and 577,000 with SAFS on the African continent in the same year. Similarly, using the pooled estimate for fungal sensitisation (23.3%), we estimate that approximately 4,074,000 adult asthmatics have fungal sensitisation in Africa.

## Discussion

The results of this systematic review indicate that data are scanty about fungal asthma and its associated complications from the African continent. Asthma is one of many neglected diseases in Africa. If antifungal therapy with oral azoles were routinely given to adults with problematic fungal asthma in Africa, about 437,000–577,000 (ABPA/SAFS) asthmatics would receive therapy and at least 60% of these would benefit [63]. Given the rising mortality from asthma in the continent, this seems the most expedient path of adding to physicians’ tools to control uncontrolled asthma. Currently this is not a treatment option for fungal asthma in Africa, and requires study before being rolled out generally. Oral itraconazole, the preferred antifungal for fungal asthma, was available in at least 43% of African nations, but costly [64]. The cost varied widely from less than $1 in Uganda to $19.4 in Nigeria for a 400mg/day dose. However, the WHO recently added itraconazole on the “2017 Model List of Essential Medicines” for adults (EML) for management of selected fungal infections [65]. This gives hope that resource-constrained African nations can access itraconazole for the management of fungal asthma, as it is off patent and there are many generic suppliers.

We found only eight epidemiological studies. The remaining majority (twelve papers) were review articles that used modelling to estimate the burden of serious fungal infections in different African countries. Whether fungal sensitisation is associated with severe asthma and which factors are associated with fungal asthma are not documented in any African country. Only 24% of the countries in Africa had any estimate for fungal asthma. There was relatively even regional distribution over the continent; three countries were from North Africa, four from West Africa, three from East Africa and three from South Africa. However, there were no estimates from central Africa.

The clinical perceptions about asthma vary in different African countries. Asthma is confused with other chronic lung diseases and symptoms such as tuberculosis, smoker’s cough, smoke exposure related to wood burning stoves inside, pollution and cold weather [66]. Education and diagnostic tools for asthma are required, to prevent inappropriate therapy, including antibiotics and anti-tuberculous agents.

The prevalence of asthma is high and increasing in Africa, possibly partly attributable to urbanisation and air pollution [6,67]. A cross-sectional world health survey estimated the prevalence of asthma in Africa at 3.9% for doctor diagnosed asthma, 4.2% for clinical asthma and 7.8% for wheezing symptoms [2]. A systematic review estimating the prevalence of asthma in Africa revealed a cumulative prevalence of “asthma ever” in South Africa (53%), Egypt (26.5%), Nigeria (18.4%), Ethiopia (16.3%) and Gambia (1.9%) [6]. However, in our review, the prevalence of asthma ranged from 3.1% to 15.2% (n = 13 studies) with an average of 6%. Similarly, the prevalence of severe asthma was estimated to range from 0.2% to 1.5% with an average of 0.6%. However, there is currently lack of a standardized case definition for severe asthma in Africa. In most African settings, severe asthma is defined based on symptoms [6,68]. In addition, only Kenya [69] and South Africa [70,71] seem to have published guidelines on the management of asthma among adults and/or children. Surprisingly, we observed a gradual steady increase in the morbidity and mortality attributed to asthma in Africa among adults. All this information emphasises the point that asthma is neglected in Africa. More advocacy is needed to increase awareness about asthma and its associated complications in Africa. This may encourage screening programmes for both asthma and allergic fungal infections in this population.

*Aspergillus* species were the most prevalent association with fungal asthma in Africa. Other fungi may be important, including *Alternaria* and *Cladosporium* spp., and these fungi also need study in Africa. Despite limited published data, the prevalence of fungal sensitisation was relatively high from the cross-sectional studies identified (range: 3–52%) with an average of 28% and a pooled estimate of 23.3%. This average and pooled estimate are similar to that of *Agarwal et al* [72] who found a pooled prevalence of *Aspergillus* sensitisation of 28%. In this review, 21 studies were included between 1964 and 2008, but only one from Africa [72].

ABPA prevalence was estimated at 2.5% of adult asthmatics in the country burden articles. However, three cross-sectional studies gave estimates of 2.6%, 21.2% and 1.9% respectively [45,46,51] for ABPA. The prevalence of SAFS in adult asthmatics was only estimated in country burden papers at 3.3%. We were unable to perform a meta-analysis on the estimates of ABPA and SAFS to get pooled figures since the confidence intervals and sample sizes could not be got from the papers. The accuracy of these estimates on prevalence of ABPA and SAFS in Africa remains unclear. In a recent review article published in 2017 which discussed the accuracy of estimates of the burden of fungal infections in 43 countries, the global prevalence of fungal asthma was estimated at over ten million cases annually [73]. The review noted that “the estimates were not intended as a substitute for high quality epidemiological study or comprehensive surveillance, but do provide a rough approximation of the size of each fungal disease by country and therefore a means of comparing countries”.

In addition to the above, the lack of data on fungal asthma in Africa mighty also be largely attributed to limited availability of diagnostic tests particularly in resource-limited settings. Apart from the review articles, majority of the epidemiological studies included in the review used skin prick tests to diagnose fungal allergy. At present, diagnosis of fungal sensitisation among patients with asthma can be made either by skin prick test or fungal specific IgE immunoassays [28,32,33], with a 77% concordance between the two tests [31]. However, most of these approaches are rarely performed in resource-constrained settings. Skin prick testing is inexpensive, but serum fungal specific IgE is currently costly and needs referral to well-equipped laboratory facilities.

Only two studies used fungal specific IgE immunoassays [47,48]. Two of the studies used *Aspergillus* precipitins test [45,46]. Precipitins detection of *Aspergillus* IgG and IgM antibody is insensitive and is most useful for chronic pulmonary aspergillosis rather than ABPA, although often positive [74]. Other tests such as fungal specific IgG/ total serum IgE immunoassays and eosinophil count can contribute to diagnosis of ABPA and SAFS as proposed by Agarwal *et al* [75]. High resolution chest CT scan may be added to distinguish between “serological ABPA” and “ABPA with bronchiectasis”. However, most of these tests are costly and not available in Africa. Introducing and advocating for point-of-care tests for fungal allergy would help to solve this problem.

### Limitations

Sixty percent (12/20) of the studies included were review articles that used modelling to estimate the national burden of fungal infections in individual countries. Meta-analysis was not performed for ABPA and SAFS due to limited data. The estimates for ABPA had a very wide range.

## Conclusion

We systematically estimated the burden of fungal asthma in Africa, highlighting the gap in data and epidemiological studies. Despite limited published data on the subject, we identified a high prevalence of fungal sensitisation in this population and a lack of modern diagnostics technologies. The accuracy of the estimates on prevalence of ABPA and SAFS from reviews remains unclear. Fungal allergy is in fact a significant but possibly underestimated problem in asthma on the African continent. There is urgent need for national epidemiological studies to estimate the actual burden of fungal asthma in Africa. In addition, there is need to develop affordable and more sensitive point-of-care diagnostic tests to improve early diagnosis and encourage screening programmes for fungal asthma in Africa. More studies are also needed to explore fungal asthma among children in Africa.

## Supporting information

S1 Protocol Protocol for systematic review. (PDF)

S1 Checklist PRISMA checklist used during the systematic review and meta-analysis. (PDF)
