# Supplementary material for: Burden of fungal asthma in Africa: A systematic review and meta-analysis
Source: PLoS One. 2019 May 16;14(5):e0216568. doi: 10.1371/journal.pone.0216568 (PMC6521988; doi:10.1371/journal.pone.0216568)
Supplement: S1 Protocol — (PDF) [file pone.0216568.s001.pdf]

PROTOCOL FOR SYSTEMATIC REVIEW

Review team:

Richard Kwizera[1,6], Joseph Musaazi[1], Freddie Bwanga[2], William Worodria[3,4], David B. Meya[1,3], Henry Kajumbula[2], Stephen J. Fowler[5], Bruce J. Kirenga[3,4,6], David W. Denning[8], Robin Gore[7],

AFFILIATIONS

1) Infectious Diseases Institute, College of Health Sciences, Makerere University, Kampala, Uganda. **2)** Department of Medical Microbiology, School of Biomedical Sciences, College of Health Sciences, Makerere University Kampala, Uganda. **3)** Department of Medicine, School of Medicine, College of Health Sciences, Makerere University, Kampala, Uganda. **4)** Mulago National Referral Hospital, Kampala, Uganda. **5)** Division of Infection, Immunity and Respiratory Medicine, School of Biological Sciences, Faculty of Biology, Medicine and Health, The University of Manchester; NIHR Biomedical Research Centre, Manchester University Hospitals NHS Foundation Trust, United Kingdom. **6)** Makerere University Lung Institute, College of Health Sciences, Makerere University, Kampala, Uganda. **7)** Cambridge University Hospitals NHS Foundation Trust, Cambridge, United Kingdom **8)** The National Aspergillosis Centre, Wythenshawe Hospital, The University of Manchester, Manchester Academic Health Science Centre, Manchester, United Kingdom

Type of systematic review:

This will be a Quantitative-meta analysis

Title of review:

Burden of fungal asthma in Africa: a systematic review and meta-analysis

Aim of the Review:

This study aims to estimate the burden of allergic fungal diseases (fungal sensitization, ABPA and SAFS) in bronchial asthma in Africa using a systematic review.

Introduction

Bronchial asthma is one of the neglected diseases in Africa with a surprisingly high prevalence. Allergic pulmonary fungal diseases especially those caused by *Aspergillus* species have been reported to complicate asthma progression and treatment outcomes. However, despite published work about allergic fungal asthma, data on its burden and associated factors remains scanty in Africa. The aim of this study will be to systematically review literature on the burden of allergic fungal asthma in Africa to highlight the gap; noting information such as prevalence, diagnosis, treatment and the effect of fungal sensitisation to the severity of asthma.

Research questions:

- What is the prevalence of fungal sensitization, ABPA and SAFS in asthma in Africa?
- What factors are associated with allergic fungal asthma in Africa?
- What is the diagnostic criteria for allergic fungal asthma in Africa?
- What treatment options are available for allergic fungal asthma in Africa?
- What is the effect of fungal sensitisation to the severity of asthma in Africa?

Population: asthma patients

Scope: Africa

Study designs

We shall aim to include all studies of any design focusing on fungal sensitisation in asthma (fungal asthma) in any African country.

Inclusion and exclusion criteria

We shall include all studies highlighting information such as prevalence, diagnosis, treatment and the effect of fungal sensitisation to the severity of asthma. We will restrict the languages to English and French since they are the main national languages in Africa. There will be no restriction to year of publication. We shall exclude all case reports/ series, studies about fungal sensitisation in other patients' populations other than asthma, studies done outside Africa and studies done in animal models. For this review, we shall define fungal sensitisation as a positive fungal specific skin prick test (SPT) or an elevated fungal specific IgE antibody titre.

Databases:

PubMed, HINARI and google scholar.

Key search words:

- Africa
- Fungal sensitisation
- aspergillus sensitisation
- fungal allergy
- fungal infections
- fungal asthma
- severe asthma with fungal sensitisation (SAFS)
- allergic bronchopulmonary aspergillosis (ABPA)
- severe asthma
- burden of fungal infections
- African country names

Search strategy

We shall perform a PubMed electronic search to identify primary studies addressing fungal asthma in Africa. In the first search, we shall use the term "Africa" together with other individual key words, such as, fungal sensitisation; aspergillus sensitisation; fungal allergy; fungal infections; fungal asthma; severe asthma with fungal sensitisation (SAFS); allergic bronchopulmonary aspergillosis (ABPA); severe asthma and burden of fungal infections.

In the second search, we shall replace the word “Africa” with specific names for each of the individual 54 African nations but keep all the other key words. Furthermore, we will repeat these two searches in HINARI and google scholar to provide more references.

Review of studies

A database will be created from the electronic searches and kept in EndNote X7 programme while restricting entry of duplicate citations. Two reviewers will screen the citations using title and abstract without blinding to capture relevant studies. The database will then be screened again using full text for each study to include only primary relevant articles. We shall only include studies addressing the burden of fungal sensitisation, fungal asthma, SAFS or ABPA in patients with bronchial asthma in any African country.

Data summary and analysis

Data from the final studies will be summarised in an excel spreadsheet, recording information such as; title, first author, year of publication, country, study type, sample size, population, prevalence of fungal sensitisation, prevalence of ABPA, prevalence of SAFS, diagnosis of fungal allergy, factors associated with fungal allergy, fungal allergy vs severity of asthma and treatment of fungal allergy. This will later be transferred to STATA for meta-analysis. Data will be analyzed using STATA version 14. Statistical/ meta-analysis will aim at determining pooled prevalence of fungal sensitization, ABPA and SAFS in bronchial asthma among Africans. We plan to calculate proportions with 95% confidence intervals (CIs) for each study and then pool the data to derive a pooled proportion with 95%CI.

Timeframe

The review is expected to take 12 months to complete.
